# Upgrading delivery rooms in Africa’s primary healthcare systems: a combination strategy of the ‘staff, stuff, space, and systems’ framework and emerging technologies

**DOI:** 10.7189/jogh.15.03006

**Published:** 2025-03-28

**Authors:** Yuxuan Li, Rudong Zhang, Ruolin Zhang, Nicholas Peoples, Chunshan Zhao, Min Yang, Kun Tang

**Affiliations:** 1 Vanke School of Public Health, Tsinghua University, Beijing, China; 2 School of Basic Medical Sciences, Tsinghua University, Beijing, China; 3 Harvard T.H. Chan School of Public Health, Harvard University, Boston, Massachusetts, USA; 4 Baylor College of Medicine, Houston, Texas, USA; 5 School of Public Health, Peking University, Beijing, China; 6 Bill and Melinda Gates Foundation China Office, Beijing, China

## Abstract

Maternal health disparities in sub-Saharan Africa remain critical, with high maternal mortality ratio requiring urgent interventions. In this viewpoint, we propose an integrated strategy combining the ‘staff, stuff, space, and systems’ (4S) framework with emerging technologies to upgrade delivery rooms in primary health care settings. Infrastructure enhancements, point-of-care innovations like artificial intelligence-driven diagnostics and ultrasound, and workforce training through traditional training-of-trainers approach or emerging simulation-based education aim to improve maternal and neonatal health outcomes. While financial and systemic barriers persist, sustainable funding, community engagement, and policy support are crucial for success. The integrated strategy offers a scalable solution to reduce maternal mortality and advance the maternal and child health targets of the 2030 Sustainable Development Goals.

## THE URGENT CHALLENGE OF MATERNAL AND CHILD HEALTH IN SUB-SAHARAN AFRICA

Tackling inequalities in maternal and child health is an urgent global health priority. Particularly alarming is the situation in sub-Saharan Africa, where in 2020, 70% of global maternal deaths occurred, which represents this region with the highest maternal mortality ratio (MMR) (545 per 100 000 live births) in the world [1]. The highest ratio not only highlights the acute risks faced by mothers in these areas but also marks a serious disparity in maternal and child health between sub-Saharan Africa and other regions. The disparity positions Africa as a key focus for catching up with the Sustainable Development Goals (SDGs) maternal and child health targets by 2030 [1], which ambitiously target an MMR of less than 70 per 100 000. To distinctly demonstrate the disparities in maternal health between Africa and the global context, we conducted a comparative analysis of selected World Health Organization (WHO) recommendations against the current realities in Africa (Table S1 in the **Online Supplementary Document****)**. The broad repercussions of maternal mortality extend beyond individual families to affect community well-being and national economic stability, even perpetuating cycles of poverty and deteriorating public health [2]. Therefore, it is imperative to implement effective measures that can significantly ameliorate these disparities, focusing on both immediate and long-term improvements in health care access and quality, ensuring that sub-Saharan Africa is not left behind in global health advancements.

To mitigate disparities in maternal care between sub-Saharan Africa and other regions, several strategies, including the upgrading of primary health care delivery rooms, have been proposed. Strengthening the infrastructure of the primary health care system through the construction and improvement of delivery rooms is a crucial strategy for reducing MMR in low-resource areas. The practicality of this strategy is exemplified by the Ugandan government’s initiative to ‘comprehensively upgrade over 200 health centres with maternal and laboratory services’ [3]. Furthermore, previous study similarly confirms that the implementation of the WHO/United Nations International Children’s Emergency Fund ‘Every Mother Every Newborn Quality Improvement standards,’ which focus on enhancing the quality of care around delivery through a comprehensive package of interventions within health facilities, including the upgrading of delivery rooms, has demonstrated promising results in reducing newborn case-fatality rates and perinatal mortality in countries like Tanzania [4]. However, implementing health care interventions in sub-Saharan Africa faces numerous barriers at various levels of the health care system, including insufficient incentives for health care workers, infrastructural inadequacies, and socio-cultural obstacles. Additionally, the absence of a clear and scalable roadmap or a defined formula for what constitutes a '’high-quality delivery room'’ remains a significant challenge, particularly in resource-constrained health care environments. To address these issues, we preliminarily propose an innovative strategy that integrates the traditional health care system improvement framework with emerging technologies.

## INNOVATIVE INTEGRATION STRATEGY FOR UPGRADING DELIVERY ROOMS IN LOW-RESOURCE REGIONS

**Figure 1:**
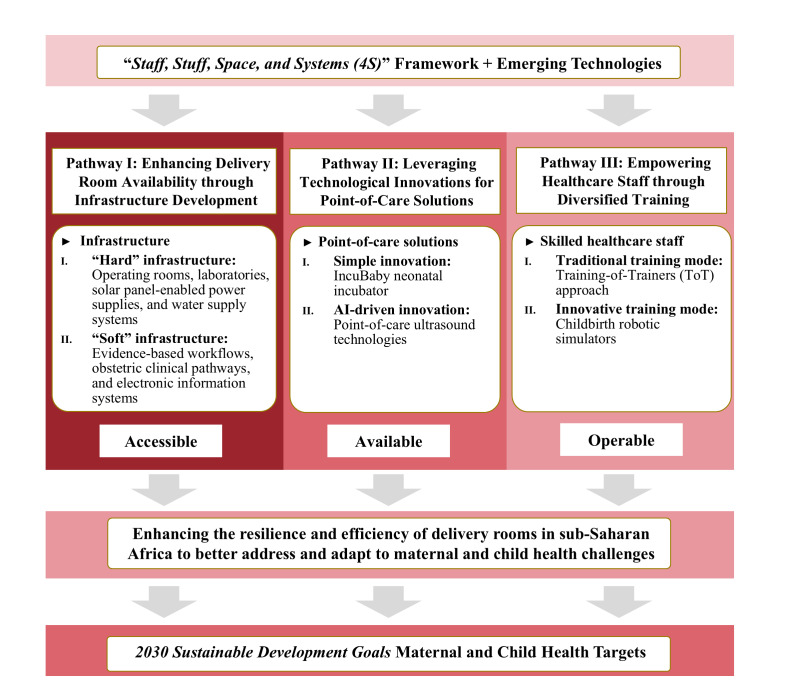
Integration strategy for upgrading delivery rooms in primary health care systems.

The integration of the ‘staff, stuff, space, and systems’ (4S) framework [5] with emerging technologies such as solar power systems, point-of-care diagnostics, and artificial intelligence [6] would offer significant advantages for enhancing the primary health care system. The combination leverages the strengths of the 4S framework–well-trained staff, reliable medical stuff, functional and safe spaces, and efficient systems to build robust health care environments. By incorporating innovative technologies, primary health care systems can become more responsive and adaptable. The organic synergy of the traditional health care system improvement framework with technological advancements ensures that the delivery room can meet current demands while also being prepared for future challenges, leading to improved maternal and child outcomes and more efficient health care delivery. Therefore, we propose an integrated strategy that combines the 4S framework with disruptive emerging technologies to create health care infrastructure. Specifically, in the context of making delivery rooms more effective, we advocate a tripartite approach that focuses on: 1) available via ‘hard’ and ‘soft’ infrastructure, 2) accessible via innovative, cost-effective point-of-care interventions, and 3) operable via an adequately sized and competent health care workforce (**Figure 1**).

### Pathway one: enhancing delivery room availability through infrastructure development

By optimising stuff, space, and systems of delivery rooms through the provision of ‘hard’ and ‘soft’ infrastructure, high-quality maternal health care can be delivered to women in nearby communities, rather than relying on distant tertiary health care centres.

Strengthening the ‘hard’ infrastructure of primary health care settings can substantially improve maternal, perinatal outcomes because it directly enhances the availability, reliability, and capability of health care services. In Tanzania, enhancing rural health centres by adding operating rooms, laboratories, solar power, and water systems led to a 151% rise in institutional deliveries, a drop in obstetric referrals from 9% to 3%, and notable reductions in maternal and neonatal mortalities [7]. Therefore, the enhancement of ‘hard’ infrastructure, including the introduction of standardised operating rooms and laboratories, allows for comprehensive onsite medical procedures and avoids crucial care delays by reducing the necessity for referrals to distant hospitals.

Incorporating ‘soft’ infrastructure into delivery room design, such as evidence-based workflows, internationally recognised clinical pathways, and electronic information systems, can create a more efficient environment. The successful introduction of case management and decision support applications in Nigeria, which improved antenatal care quality scores from 13.3 to 17.2, proves that evidence-based clinical decision-making can be facilitated by mHealth [8]. The urgent need for ‘soft’ infrastructure is further highlighted by a systematic review of MMR in sub-Saharan Africa, which identifies the lack of data sets and government reports and recommends establishing health information systems to effectively identify and analyse the causes of maternal mortality [9]. Within the ‘soft’ infrastructure, feedback loops could also be established to monitor the effectiveness of delivery rooms and make necessary adjustments.

Meanwhile, enhancing health care infrastructure in sub-Saharan Africa depends on strong community engagement and sustainable financing models, which build trust and support long-term investments. Overcoming challenges like financial instability and socio-cultural resistance is essential to fully realise the potential of these advancements.

### Pathway two: leveraging technological innovations for point-of-care solutions

Leveraging innovation has considerable potential in maternal primary health care. When intelligently integrated into the primary health care system, point-of-care interventions can decentralise and enhance diagnostic and treatment capabilities cost-effectively, thereby transforming the mode of service delivery.

A prime example is the IncuBaby neonatal incubator, which can prevent neonatal hypothermia in low-resource environments by using electric blankets and a temperature probe to continuously self-regulate the temperature of the incubator [10]. Notably, delays in vulnerable newborns receiving high-quality care are one of the reasons for the persistently high neonatal mortality rates in Africa [11]. The incubator, which allows health care providers to deliver immediate, life-saving interventions right at the point of care with autonomous regulation and without requiring extensive additional training or resources, could be an excellent solution to this issue. More ambitiously, the Bill and Melinda Gates Foundation supports the development of artificial intelligence-driven, point-of-care ultrasound technologies as an emerging antenatal care tool. This technology has the potential to make obstetric ultrasound screening accessible to pregnant women affordably and sustainably [12]. Thus, innovative solutions not only advance the quality of care but expand accessibility to ensure that essential health care services reach those most in need, regardless of geographical and economic barriers.

To ensure the long-term sustainability of technical interventions for maternal primary health care, it requires financial support, regular maintenance, and ongoing staff training. Addressing the constraints of limited resources and the need for local expertise will further bolster the effectiveness of these interventions.

### Pathway three: empowering health care staff through diversified training

Addressing the critical aspect of ‘staff.’ It becomes evident that no matter how well-equipped delivery rooms are, their effectiveness is largely associated with the presence of a competent health care workforce. Research by Nove and colleague*s* offers a striking estimate that up to 67% of maternal deaths, 64% of newborn deaths, and 65% of stillbirths could be prevented in 88 low- and middle-income countries (LMICs) by the provision of qualified midwives practising in multidisciplinary teams [13]. The skilled health care workforce would save an estimated 4.3 million lives annually by 2035, and scholars advocated that these findings ‘should command the attention of the global community in the same way that a new drug or innovative technical intervention would’ [14]. Despite the clear benefits, there remains a significant gap in global, regional, and national planning, where the enormous potential contribution of a skilled midwifery workforce is frequently underestimated [14]. Thus, there is also a growing need to invest in the development and maintenance of a robust, adequately sized, and competent maternal clinical workforce to ensure that delivery rooms are skilfully managed.

In LMICs, the effectiveness of the training-of-trainers approach has been well-documented, as evidenced by the significant knowledge transfer achieved in managing pre-eclampsia and eclampsia in Nigeria [15]. The adaptability of the training-of-trainers approach to feedback ensures its relevance over time and across different trainer cohorts, making it a sustainable approach for long-term training programmes [16]. Building on this, we believe that combining high-fidelity modelling with on-site training focused on human factors provides the health care workforce with a safe environment to practice both technical and non-technical skills, which are essential for improving maternal and neonatal outcomes [17]. As technology advances, we anticipate the transfer of advanced training techniques from high-income countries to LMICs. Specialised training, such as using childbirth robotic simulators, can provide midwives with educational experiences across various delivery scenarios, thereby enhancing the operational efficiency of delivery rooms [18]. This comprehensive, reality-based training ensures that the health care workforce is not only technically proficient but also demonstrates enhanced leadership capabilities, thus promoting continuous professional development.

Empowering health care staff through training and international collaboration is crucial for improving maternal health services. While political instability and bureaucratic delays present challenges, proactive strategies and continued support can mitigate these obstacles, ensuring the progress of health care improvements.

## FACILITATORS AND BARRIERS ANALYSIS OF THE PROPOSED STRATEGY

The integration of the 4S framework with emerging technologies to upgrade delivery rooms in sub-Saharan Africa holds significant promise for reducing disparities in global maternal and child health. However, the successful implementation of these interventions requires a comprehensive understanding of both facilitators and barriers across three key pathways (Figure S1 in the **Online Supplementary Document**).

Facilitators for enhancing health care infrastructure include strong community engagement, which fosters trust and ensures that health care services are culturally sensitive, ultimately increasing service utilisation. For example, in Mozambique, community engagement strategies have successfully incorporated maternal health-promotion activities into routine care, significantly improving care-seeking behaviours and emergency preparedness [19]. Sustainable financing mechanisms, such as government-community co-financing models successfully implemented in Nigeria, also play a critical role in supporting the long-term costs of health care infrastructure and services [20]. To sustain technical interventions in maternal health care, regular maintenance protocols are essential to keep equipment functional and reliable. In addition, continuous training programmes for health care workers and technical staff could ensure they can operate new technologies efficiently and adapt to evolving advancements, thereby improving health care outcomes.

However, despite these facilitators, several barriers persist that could impede the success of these initiatives. Resource limitations – especially a lack of financial support – are a major challenge, as long-term investments in infrastructure are often constrained by limited and unstable funding. The financial instability also creates difficulties in acquiring and maintaining advanced technologies. Additionally, resistance to new practices and inadequate incentives slow progress. Patient-public engagement in sub-Saharan Africa remains low due to socio-cultural factors and distrust in the health care system, further complicating the implementation of universal health coverage [21]. A shortage of trained staff, poor incentives, and resistance to change compound these challenges [22]. Furthermore, political instability and bureaucratic delays often disrupt or hinder the establishment of critical training facilities and programmes.

## THE FUTURE PATH AND ASPIRATIONS FOR GLOBAL MATERNAL AND CHILD HEALTH

Achieving the SDG maternal and child health targets by 2030 requires urgent and intensified action in Africa. As presented in Nature Medicine, the development of comprehensive strategies – such as increasing facility births, improving the availability and quality of clinical services, and incorporating community-based interventions – has shown promising results, with the MMR projected to drop to 58 per 100 000 live births by 2030, based on the analysis covering the duration between 2022–30 [23]. It can be seen that upgrading maternal health care services in Africa’s primary health care systems will be a firm step toward remediating these extreme maternal disparities. By applying the 4S framework and emerging technologies, we can initially optimise these delivery rooms to enhance their availability, accessibility, and functionality. In the near future, we anticipate the emergence of high-quality case studies that will rigorously evaluate and validate the impact of the integrated strategy of the 4S framework and emerging technologies discussed in this viewpoint. Such studies are expected to provide critical insights into how these pathways affect mortality rates and related health outcomes. Doing so offers a promising strategy to help move the needle from merely aspiring to the 2030 SDG targets to actively working towards achieving them.

## Additional material


Online Supplementary Document
