# Supplementary material for: Upgrading delivery rooms in Africa’s primary healthcare systems: a combination strategy of the ‘staff, stuff, space, and systems’ framework and emerging technologies
Source: J Glob Health. 2025 Mar 28;15:03006. doi: 10.7189/jogh.15.03006 (PMC11949509; doi:10.7189/jogh.15.03006)
Supplement: Online Supplementary Document [file jogh-15-03006-s001.pdf]

Supplement to: Li Y, Zhang R, Zhang R, Peoples N, Zhao C, Yang M, Tang K. Upgrading delivery rooms in Africa's primary healthcare systems: a combination strategy of the 'staff, stuff, space, and systems' framework and emerging technologies. J Glob Health. 2025;15:03006.

Figure S1. Facilitators and barriers analysis of proposed strategy.

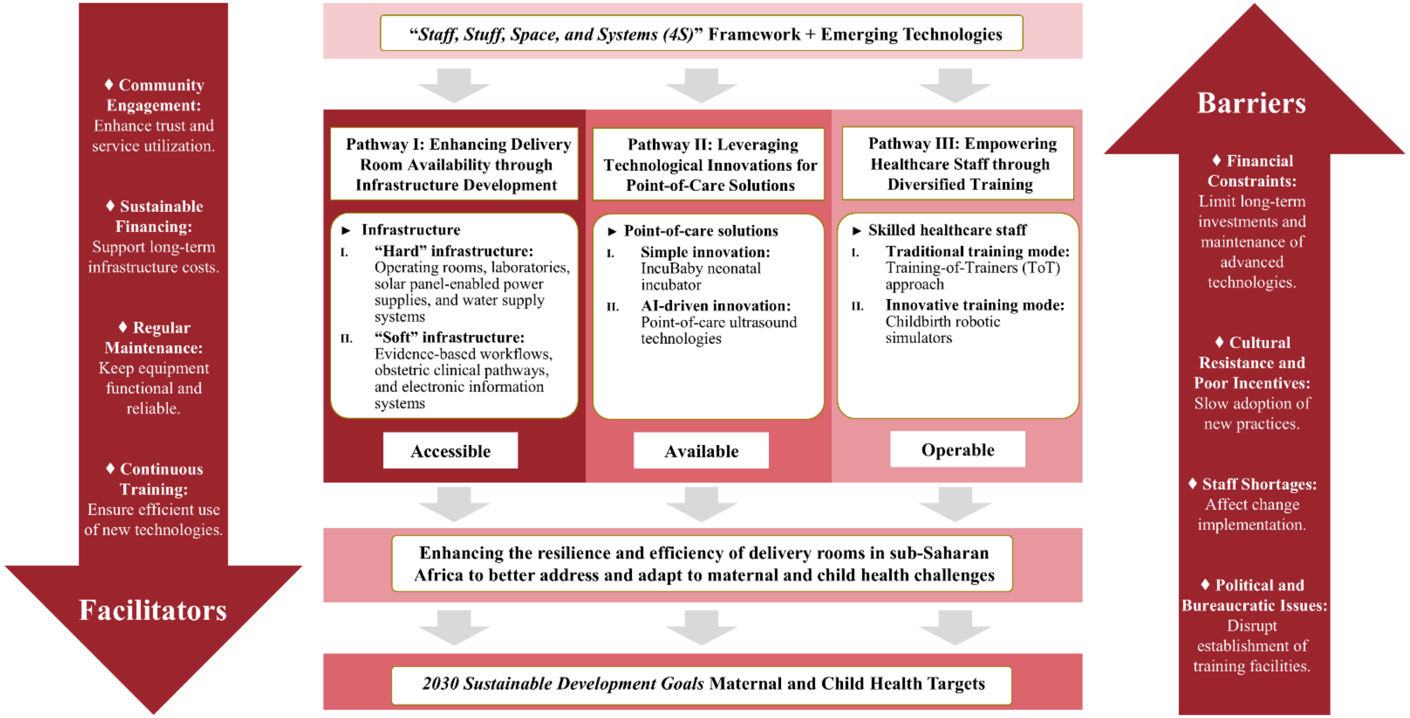

Figure 2 Facilitators and Barriers Analysis of Proposed Strategy

Supplementary Table 1. Antenatal, childbirth, postpartum, and newborn care: comparing *WHO* recommendations with African contexts

	WHO Recommendations	African Contexts	Influence Factors
**Antenatal**	♦ **Quality care:** Ensuring that women receive proper medical attention during pregnancy [1].	♦ **Quality care:** Only 53.8% of women across 13 sub-Saharan Africa (SSA) countries receive proper antenatal services, with significant country variations [2].	♦ **Quality care:** Education level, marital status, number of antenatal care visits, and community factors [3].
	♦ **Increasing antenatal care (ANC) contacts:** Moving from 4 to 8 visits for better pregnancy outcomes [1].	♦ **Increasing antenatal care (ANC) contacts:** Only 6.8% of women in SSA achieving 8 ANC contacts [4].	♦ **Increasing antenatal care (ANC) contacts:** Educational inequalities and limited healthcare infrastructures [4].
	♦ **Timely and adequate visits:** Ensuring pregnant women receive care at critical times [1].	♦ **Timely and adequate visits:** Just 41.2% receive care at the right time, and only 10.4% get sufficient attention [2].	♦ **Timely and adequate visits:** Economic status, education, and access to health insurance [2].
**Childbirth**	♦ **Skilled birth attendants (SBA):** Ensuring trained professionals assist during deliveries [5].	♦ **Skilled birth attendants (SBA):** The coverage of SBA in sub-Saharan Africa varies widely, ranging from 24.3% in Chad to 96.7% in South Africa, with significant wealth-related inequalities across countrie [6].	♦ **Skilled birth attendants (SBA):** Cultural preferences and cost concerns. Proper training can enhance outcomes [7].
	♦ **Emergency obstetric care:** Getting care within 2 hours to prevent life-threatening complications [8].	♦ **Emergency obstetric care:** At least a third of women cannot reach their nearest emergency obstetric care facility within the recommended two-hour timeframe [9].	♦ **Emergency obstetric care:** Poor road conditions, complex terrain [9] and lack of equipment [10] are important factors affecting emergency obstetric care.
	♦ **Respectful care:** Promoting a supportive environment during childbirth [11].	♦ **Respectful care:** Only 40.5% of women in Southern Ethiopia experience dignified and considerate care [12].	♦ **Respectful care:** Planned pregnancies, facility-based deliveries, family support, and awareness of available services [12].
**Postpartum**	♦ **24-hour care and checkups:** Providing high-quality care for 24 hours after	♦ **24-hour care and checkups:** 71.7% of women in sub-Saharan Africa receive	♦ **24-hour care and checkups:**

	delivery, plus three postnatal checkups in the first six weeks [13].	postpartum checkups, but disparities in care persist [14].	Education level, wealth, media exposure, and distance to medical institutions [15].
	♦ **Maternal death reviews (MDR):** Analyzing maternal deaths to improve care systems [16].	♦ **Maternal death reviews (MDR):** In Ethiopia, the Maternal Death Surveillance and Response (MDSR) system was introduced to track maternal deaths, but politicization hampers accurate and complete data collection [17].	♦ **Maternal death reviews (MDR):** Delayed referrals, missed diagnoses, low drug stocks [18], quality of data collection and reporting [19].
**Newborn**	♦ **Breastfeeding:** Advocating for exclusive breastfeeding to support infant health [13].	♦ **Breastfeeding:** Only 37% of infants under six months were exclusively breastfed, below the WHO target of 50% by 2025 [20].	♦ **Breastfeeding:** HIV risks, lack of supportive workplace policies [21].
	♦ **Newborn health assessments:** Regular checkups to monitor and ensure healthy development [22].	♦ **Newborn health assessments:** Just 23.51% of newborns receive sufficient postnatal attention [23].	♦ **Newborn health assessments:** Insufficient medical equipment, consumables, health system infrastructure [24], and poor attitude of medical staff [25].
	♦ **Kangaroo Mother Care (KMC):** Skin-to-skin contact and breastfeeding for low-birth-weight infants [26].	♦ **Kangaroo Mother Care (KMC):** While KMC was beneficial, it was perceived as a foreign concept, and cultural practices posed challenges to its acceptance [27].	♦ **Kangaroo Mother Care (KMC):** Cultural sensitivity and community involvement [27], lack of clear evidence of cost-effectiveness and safety [28].

References:

1. World Health Organization. WHO Recommendations on Antenatal Care for a Positive Pregnancy Experience: Summary. Geneva, Switzerland: World Health Organization (WHO), USAID (United States Agency for International Development); 2018. Report No.: WHO/RHR/18.02.
2. Habte A, Tamene A, Melis T. Compliance towards WHO recommendations on antenatal care for a positive pregnancy experience: Timeliness and adequacy of antenatal care visit in Sub-Saharan African countries: Evidence from the most recent standard Demographic Health Survey data. PloS One. 2024;19(1):e0294981.
3. Ameyaw EK, Baatiema L, Naawa A, Odame F, Koramah D, Arthur-Holmes F, Frimpong SO, Hategeka C. Quality of antenatal care in 13 sub-Saharan African countries in the SDG era: evidence from Demographic and Health Surveys. BMC Pregnancy Childbirth. 2024 Apr 23;24(1):303.
4. Tessema ZT, Tesema GA, Yazachew L. Individual-level and community-level factors associated with eight or more antenatal care contacts in sub-Saharan Africa: evidence from 36 sub-Saharan African countries. BMJ Open. 2022 Mar 10;12(3):e049379.
5. World Health Organization, International Confederation of Midwives, International Federation of Gynecology and Obstetrics. Making pregnancy safer: the critical role of the skilled attendant. Geneva, Switzerland: World Health Organization (WHO), International Confederation of Midwives (ICM), International Federation of Gynecology and Obstetrics (FIGO); 2004. Report No.: ISBN 9241591692.
6. Bobo FT, Asante A, Woldie M, Dawson A, Hayen A. Spatial patterns and inequalities in skilled birth attendance and caesarean delivery in sub-Saharan Africa. BMJ Glob Health. 2021 Oct 1;6(10):e007074.
7. Garces A, McClure EM, Espinoza L, Saleem S, Figueroa L, Bucher S, Goldenberg RL. Traditional Birth Attendants and Birth Outcomes in Low-Middle Income Countries: A Review. Semin Perinatol. 2019 Aug;43(5):247-51.
8. World Health Organization. Ending Preventable Maternal Mortality (EPMM): A Renewed Focus for Improving Maternal and Newborn Health and Wellbeing. World Health Organization (WHO), Maternal Health (MAH) Team, Maternal, Newborn, Child & Adolescent Health & Ageing (MCA); 2021. Report No.: ISBN: 9789240040519.
9. Banke-Thomas A, Wright K, Collins L. Assessing geographical distribution and accessibility of emergency obstetric care in sub-Saharan Africa: a systematic review. J Glob Health. 9(1):010414.
10. Okonofua F, Yaya S, Owolabi T, Ekholuenetale M, Kadio B. Unlocking the Benefits of Emergency Obstetric Care in Africa. Afr J Reprod Health. 2016 Mar;20(1):9-15.
11. Vallely LH, Shalit A, Nguyen R, Althabe F, Pingray V, Bonet M, Armari E, Bohren M, Homer C, Vogel JP. Intrapartum care measures and indicators for monitoring the implementation of WHO recommendations for a positive childbirth experience: a scoping review. BMJ Open. 2023 Nov 22;13(11):e069081.
12. Utalo D, Israel E, Lenjebo TL, Aynalem A, Darebo TD. Determinants of respectful maternity care among women who gave childbirth in Southern Ethiopia. BMC Health Serv Res. 2024 Apr 10;24:451.
13. World Health Organization. WHO Recommendations on Maternal and Newborn Care for a Positive Postnatal Experience. Geneva, Switzerland: World Health Organization; Report No.: ISBN 978-92-4-004598-9.
14. Benova L, Owolabi O, Radovich E, Wong KLM, Macleod D, Langlois EV, Campbell OMR. Provision of postpartum care to women giving birth in health facilities in sub-Saharan Africa: A cross-sectional study using Demographic and Health Survey data from 33 countries. PLOS Med. 2019 Oct 23;16(10):e1002943.
15. Dickson K, Ayebeng C, Adu-Gyamfi A, Okyere J. Postnatal care service utilisation for babies within the first two months after childbirth: an analysis of rural-urban differences in eleven Sub-Saharan African countries. BMC Pregnancy Childbirth. 2023 Jun 7;23.
16. World Health Organization. Time to Respond: A Report on the Global Implementation of Maternal Death Surveillance and Response. Geneva, Switzerland: World Health Organization (WHO); 2016. Report No.: ISBN 9789241511230.
17. Melberg A, Mirkuzie AH, Sisay TA, Sisay MM, Moland KM. 'Maternal deaths should simply be 0': politicization of maternal death reporting and review processes in Ethiopia. Health Policy Plan. 2019 Sep 1;34(7):492-8.
18. Jean-Bertin Bukasa K, Mataka A, Chongo G, Kamavu L, Chola P, Manyando C, Debrouwere V, Ippolito M. Impact of maternal death reviews at a rural hospital in Zambia: A mixed methods study. Int J Equity Health. 2020 Jul 9;19.
19. Said A, Pembe AB, Massawe S, Hanson C, Målqvist M. Maternal death surveillance and response in Tanzania: comprehensiveness of narrative summaries and

action points from maternal death reviews. BMC Health Serv Res. 2021 Jan 11;21.
20. Bhattacharjee NV, Schaeffer LE, Marczak LB, Ross JM, Swartz SJ, Albright J, Gardner WM, Shields C, Sligar A, Schipp MF, Pickering BV, Henry NJ, Johnson KB, Louie C, Cork MA, Steuben KM, Lazzar-Atwood A, Lu D, Kinyoki DK, Osgood-Zimmerman A, Earl L, Mosser JF, Deshpande A, Burstein R, Woyczynski LP, Wilson KF, VanderHeide JD, Wiens KE, Reiner RC, Piwoz EG, Rawat R, Sartorius B, Davis Weaver N, Nixon MR, Smith DL, Kassebaum NJ, Gakidou E, Lim SS, Mokdad AH, Murray CJL, Dwyer-Lindgren L, Hay SI. Mapping exclusive breastfeeding in Africa between 2000 and 2017. Nat Med. 2019 Aug;25(8):1205-12.
21. Doherty T, Horwood C, Haskins L, Magasana V, Goga A, Feucht U, Sanders D, Tylleskar T, Kauchali S, Dhansay MA, Rollins N, Kroon M, Engebretsen IMS. Breastfeeding advice for reality: Women’s perspectives on primary care support in South Africa. Matern Child Nutr. 2020 Jan;16(1):e12877.
22. World Health Organization. WHO Recommendations on Newborn Health: Guidelines Approved by the WHO Guidelines Review Committee. Geneva, Switzerland: World Health Organization (WHO); 2017. Report No.: WHO/MCA/17.07.
23. Eshetu HB, Aragaw FM, Negash WD, Belachew TB, Asmamaw DB, Tareke AA, Asratie MH. Assessing postnatal care for newborns in Sub-Saharan Africa: A multinational analysis. PLOS ONE. 2024 Feb 15;19(2):e0298459.
24. Griffin J, Jobe A, Rouse D, McClure E, Goldenberg R, Kamath-Rayne B. Evaluating WHO-Recommended Interventions for Preterm Birth: A Mathematical Model of the Potential Reduction of Preterm Mortality in Sub-Saharan Africa. Glob Health Sci Pract. 2019 Jun 1;7:215-27.
25. Manu A, ten Asbroek G, Soremekun S, Gyan T, Weobong B, Tawiah-Agyemang C, Danso S, Amenga-Etego S, Owusu-Agyei S, Hill Z, Kirkwood B. Evaluating the implementation of community volunteer assessment and referral of sick babies: lessons learned from the Ghana Newhints home visits cluster randomized controlled trial. Health Policy Plan. 2014 Sep 1;29 Suppl 2:ii114-27.
26. World Health Organization. WHO Recommendations on Interventions to Improve Preterm Birth Outcomes. Geneva, Switzerland: World Health Organization (WHO), Guidelines Review Committee, Maternal, Newborn, Child & Adolescent Health & Ageing (MCA), Sexual and Reproductive Health and Research (SRH); 2015. Report No.: ISBN 978 92 4 150898 8.
27. Mpongwana-Ncetani S, Roomaney R, Lachman A. Experiences of Xhosa women providing Kangaroo mother care in a tertiary hospital in the Western Cape, South Africa. South Afr J Psychol. 2023 Dec 1;53(4):497-508.
28. Tumukunde V, Medvedev MM, Tann CJ, Mambule I, Pitt C, Opondo C, Kakande A, Canter R, Haroon Y, Kirabo-Nagemi C, Abaasa A, Okot W, Katongole F, Ssenyonga R, Niombi N, Nanyunja C, Elbourne D, Greco G, Ekirapa-Kiracho E, Nyirenda M, Allen E, Waiswa P, Lawn JE, Mutumba R, Nambuya H, Nayiga I, Nyanzi M, Sherine OS, Nabawanuka D, Anguparu M, Batani A, Bingi G, Byaruhanga E, Dauda M, Nathan O, Peterson K, Yayi A, Seeley J. Effectiveness of kangaroo mother care before clinical stabilisation versus standard care among neonates at five hospitals in Uganda (OMWaNA): a parallel-group, individually randomised controlled trial and economic evaluation. The Lancet. 2024 Jun 8;403(10443):2520-32.
